# [Retracted] Slug contributes to cancer progression by direct regulation of ERα signaling pathway

**DOI:** 10.3892/ijo.2024.5689

**Published:** 2024-09-09

**Authors:** Youqiang Li, Yanyuan Wu, Thomas C. Abbatiello, Warren L. Wu, Ju Ri Kim, Marianna Sarkissyan, Suren Sarkissyan, Seyung S. Chung, Yahya Elshimali, Jaydutt V. Vadgama

Int J Oncol 46: 1461-1472, 2015; DOI: 10.3892/ijo.2015.2878

Following the publication of the above paper, it was drawn to the Editor's attention by concerned readers that β-actin bands shown in Figs. 1, 2 and 4 were strikingly similar, where the experimental conditions reported in Fig. 4 differed from those in Figs. 1 and 2; moreover, the Slug protein bands featured in Figs. 4a and 5a were remarkably similar in spite of the different experimental conditions that were reported in the respective figure legends, and the shape of the vimentin protein bands in Fig. 5e bore a strong similarity to the Slug protein bands that were featured in Fig. 2c, in spite of the bands being of slightly different sizes and arranged in a different orientation.

Although the possibility of publishing a corrigendum was considered, software analysis of the highlighted bands performed independently by the Editorial Office demonstrated that the bands in question were likely to have been matching bands. Therefore, given the number of potential concerns that were identified with the assembly of various of the figures in this paper, the Editor of *International Journal of Oncology* has decided not to proceed with a corrigendum, and has determined that the paper should instead be retracted from the Journal on account of an overall lack of confidence in the originally presented data. The authors were asked for an explanation to account for these concerns, but the Editorial Office did not receive a satisfactory reply. The Editor apologizes to the readership for any inconvenience caused.

